# Changed Rumen Fermentation, Blood Parameters, and Microbial Population in Fattening Steers Receiving a High Concentrate Diet with *Saccharomyces cerevisiae* Improve Growth Performance

**DOI:** 10.3390/vetsci8120294

**Published:** 2021-11-28

**Authors:** Kampanat Phesatcha, Burarat Phesatcha, Krittika Chunwijitra, Metha Wanapat, Anusorn Cherdthong

**Affiliations:** 1 Department of Animal Science, Faculty of Agriculture and Technology, Nakhon Phanom University, Nakhon Phanom 48000, Thailand; 2 Department of Agricultural Technology and Environment, Faculty of Sciences and Liberal Arts, Rajamangala University of Technology Isan, Nakhon Ratchasima 30000, Thailand; Burarat_kat@hotmail.co.th; 3 Department of Food Technology, Faculty of Agriculture and Technology, Nakhon Phanom University, Nakhon Phanom 48000, Thailand; krittika@npu.ac.th; 4 Tropical Feed Resources Research and Development Center (TROFREC), Department of Animal Science, Faculty of Agriculture, Khon Kaen University, Khon Kaen 40002, Thailand; metha@kku.ac.th

**Keywords:** fattening steers, growth performance, rumen ecology, yeast

## Abstract

The effect of dry yeast (DY) (*Saccharomyces cerevisiae*) supplementation in a high-concentrate diet was evaluated for rumen fermentation, blood parameters, microbial populations, and growth performance in fattening steers. Sixteen crossbred steers (Charolais x American Brahman) at 375 ± 25 kg live weight were divided into four groups that received DY supplementation at 0, 5, 10, and 15 g/hd/d using a completely randomized block design. Basal diets were fed as a total mixed ration (roughage to concentrate ratio of 30:70). Results showed that supplementation with DY improved dry matter (DM) intake and digestibility of organic matter (OM), neutral detergent fiber (NDF), and acid detergent fiber (ADF) (*p* < 0.05), but DM and crude protein (CP) were similar among treatments (*p* > 0.05). Ruminal pH (>6.0) of fattening steer remained stable (*p* > 0.05), and pH was maintained at or above 6.0 with DY. The concentration of propionic acid (C_3_) increased (*p* < 0.05) with 10 and 15 g/hd/d DY supplementation, while acetic acid (C_2_) and butyric acid (C_4_) decreased. Methane (CH_4_) production in the rumen decreased as DY increased (*p* < 0.05). *Fibrobacter succinogenes* and *Ruminococcus flavefaciens* populations increased (*p* < 0.05), whereas protozoal and methanogen populations decreased with DY addition at 10 and 15 g/hd/d, while *Ruminococcus albus* did not change (*p* > 0.05) among the treatments. Adding DY at 10 and 15 g/hd/d improved growth performance. Thus, the addition of DY to fattening steers with a high concentrate diet improved feed intake, nutrient digestibility, rumen ecology, and growth performance, while mitigating ruminal methane production.

## 1. Introduction

Fattening beef production systems enhance animal growth and profitability by feeding high-concentrate diets with minimal roughage [1]. However, the ingestion of large amounts of readily fermentable substrates increases the risk of digestive diseases such as ruminal acidosis [2,3]. To avoid acidosis, preventive measures include modifying feeding management and/or incorporating additives into the total mixed ration (TMR) [4].

Yeast products, such as *Saccharomyces cerevisiae*, have been employed as antibacterial feed additive substitutes [5]. Yeast improves the reducing environment in the rumen by aiding in the growth of cellulolytic bacteria and lactate-consuming populations, thereby improving rumen stabilization and enhancing the capability to digest fiber [6,7,8]. The composition of bovine diets varies, and the addition of live yeast affects the balance and activity of rumen microbial populations as well as feed digestion [9,10,11]. Yeast has been widely utilized as a supplement to improve digestive issues, feed intake, and efficiency in dairy calves [12]. Yeasts are known to improve ruminal microbial activity by regulating pH, particularly in calves with high dietary consumption of non-structural carbohydrates that may alter the microbial environment [13]. High-concentrate feeds also have a negative impact on cow consumption and sorting activity [14]. In ruminants, yeast supplementation improved nutrient digestibility [15], decreased methane (CH_4_) production [7,16], and improved dairy cow performance [17,18,19,20]. However, the literature has described conflicts about the influence of *S. cerevisiae* supplementation on the rumen of growing beef cattle fed a high-concentrate diet. Supplementation of active dry yeast (ADY) with a high-concentrate diet improves nutrient utilization via enhanced digestibility and rumen ecology while reducing CH_4_ production in fattening steers.

This study investigated the influence of supplementing a high concentrate diet with *S. cerevisiae* on DM, feed intake, nutritional digestibility, rumen parameters, rumen microorganisms, CH_4_ production, and growth performance in fattening steers.

## 2. Materials and Methods

The study design and plan strictly followed the norms of the Animal Ethics Committee of Nakhon Phanom University (permission No. AENPU A2/2560).

### 2.1. Animals, Feed, and Experimental Design

Sixteen crossbred steers (Charolais × American Brahman) at 375 ± 25 kg live weight were divided into four groups that received DY supplementation at 0, 5, 10, and 15 g/hd/d using a completely randomized block design. Basal diets were fed as a total mixed ration (roughage to concentrate ratio of 30:70). All cows were fed ad libitum with free access to water and mineral blocks. The brewer’s yeast strain, *S. cerevisiae,* in this study was obtained from the Renu Nakhon district, Nakhon Phanom Province, Thailand. The ingredients and chemical composition of the TMR are shown in Table 1. The animals were dewormed and given a 14-day acclimation period prior to the experiment. The trial lasted over 104 days, with the digestibility test taking place in the final week. The individual feed was made and fed in the morning and evening. To measure daily feed intake, the amount of feed supplied and denied was recorded every day. Average daily gain (ADG) was computed as the difference between the starting and final live weight, which was calculated by dividing the cumulated gain by the total fattening days. The ratio of individual DMI to dietary ADG was used to measure feed efficiency, which was calculated by dividing the average DM intake by the ADG of each fattening steer.

### 2.2. Sample Collection and Chemical Analyses of Samples

Representative feed and fecal samples were performed on the last 7 days of the experimental period. Fecal samples were obtained by rectal sampling of about 100 g. The samples were then composited by the animal and stored at −20 °C for later chemical analyses. The composite samples were dried at 60 °C before being processed (1-mm screen, Tecator, 1093, Hoganas, Sweden) and analyzed for DM, CP, and ash [21]. The acid detergent fiber (ADF) was determined and expressed, including residual ash. The neutral detergent fiber (NDF) was determined with the addition of alpha-amylase, but without sodium sulphite, and the results are given inclusive of residual ash [22]. Nutrient digestibility was calculated using acid insoluble ash (AIA) [23], and DM, OM, NDF, and ADF digestibility were determined from the ratio of AIA in feed and faeces, and digestibility of nitrogen was determined from the ratios of AIA and nitrogen (N) in feed and faeces.

Blood samples and rumen fluid were collected at 0 and 4 h after the morning feeding on the final day of the experimental period. Each time, approximately 200 mL of rumen fluid and digesta from the rumen were collected through the oral cavity using a stomach tube connected to a vacuum pump. Rumen fluid pH and temperature were immediately measured, and 50 mL of rumen fluid was collected and mixed with 5 mL of 1M H_2_SO_4_ to stop microbial activity fermentation before centrifugation at 16,000× *g* for 15 min. A total of 20 mL of supernatant was taken and frozen at −20 °C before being analyzed in the laboratory for ammonia–nitrogen (NH_3_-N) using micro-Kjeldahl methods.

High Performance Liquid Chromatography was used to examine volatile fatty acids (VFAs) in the rumen fluid samples (HPLC; Model Water 600; UV detector, Millipore Corp., Milford, MA, USA). Rumen CH_4_ production was approximated using the equation of Moss et al. [24] using VFA proportions, which are as follows: production of CH_4_ = 0.45 (acetate, C_2_) + 0.275 (propionate, C_3_) + 0.4 (butyrate, C_4_).

The second portion was frozen at −20 °C before being analyzed in the laboratory for microbial populations using real-time qPCR. Isolation of genomic DNA was conducted from rumen fluid and digesta. QIAgen DNA Mini Stool Kit columns were used to purify the DNA (QIAGEN, Valencia, CA USA). Real-time qPCR was adopted to determine the relative populations of total bacteria such as *Fibrobacter succinogenes, Ruminococcus albus, Butyrivibrio fibrisolvens*, *Ruminococcus flavefaciens, Megasphaera eldsdenii,* protozoa, and methanogen.

Isolation of genomic DNA was used in real-time quantitative PCR assays with power SYBR Green PCR Master Mix (Applied Biosystems, Warrington, UK), forward and reverse primers, and template DNA. Specific primers to measure the microbial population of total bacteria according to Edwards et al. [25], *F. succinogenes* and *R. flavefaciens* [26], *R. albus* [27], *B. fibrisolvens, M. eldsdenii* [28], methanogenic archaea [29], and protozoa [30] (Table 2). The DNA standards Real-time PCR amplification and detection were determined using a Chromo 4™ system (Bio-Rad, Hercules, CA, USA). 

Blood was drawn from all steers on the final day of the study via the jugular vein with 10 mL tubes. Tubes were centrifuged at 3200× *g* for 15 min at room temperature, and the separated serum was stored at −20 °C for measurement of urea nitrogen, free fatty acid (FFA), total cholesterol (TC), triglyceride (TG), glucose, and total protein (TP) (Refloton plus, Roche, Rotkreuz, Switzerland).

### 2.3. Statistical Methods

The data were analyzed using the MIXED procedure in the SAS software [31]. The mathematical model assumption used was:

Y_i_ = μ + T_i_ + β_i_ + ε_i_ (1)

where Y_i_ is the dependent variable, μ is the overall mean, T_i_ is the ith treatment effect (supplementation of *Saccharomyces cerevisiae* at 0, 5, 10, and 15 g/hd/day), β_i_ is the *i*th block effect, and _i_ is the residual error of the *i*th observation. Differences among means with *p* < 0.05 were represented as statistically significant differences. Orthogonal polynomials for diet responses were determined by linear and quadratic effects.

## 3. Results

### 3.1. Feed Intake, Growth Performance, and Nutrients Digestibility

Table 3 shows the effect of DY addition on DM feed intake, growth performance and nutrient digestibility of fattening steers. The results showed that DY addition enhanced final BW, ADG, and DM feed intake of total mixed ration compared to the control group (*p* < 0.05). Supplementation DY at 10–15 g/hd/d increased digestibility of OM, NDF, and ADF (*p* < 0.05), but did not change digestibility of DM and CP (*p* > 0.05). Feeding with a high concentrate diet without DY addition resulted in lower fiber digestion in the rumen and, thus, lower ruminal pH.

### 3.2. Rumen Parameters and Blood Metabolite

Table 4 shows how DY addition impacted rumen parameters and blood metabolites in fattening steers. Ruminal temperature (39.4–39.8 °C) and ruminal pH (>6.0) of fattening steer remained stable (*p* > 0.05), and pH was maintained at or above 6.0 with DY. The concentration of NH_3_–N increased in the DY supplementation groups and was highest at 10–15 g/hd/d, but did not affect the concentration of BUN, glucose, FFA, TP, TG, and TC (*p* > 0.05). 

### 3.3. Volatile Fatty Acid (VFA) Profiles and Methane (CH_4_) Production

Concentrations of total volatile fatty acid (TVFA) and butyric acid (C_4_) concentrations did not change, whereas propionic acid (C_3_) increased (*p* < 0.05) with DY supplementation, particularly for DY at 10–15 g/hd/d. However, acetic acid (C_2_) concentration, C_2_:C_3_ ratio, and CH_4_ production were reduced with the addition of DY at 10–15 g/hd/d.

### 3.4. Microbial Population 

Total bacteria such as *F. succinogenes*, *B. fibrisolvens, R. flavefaciens,* and *M. eldinii* increased, whereas protozoa and methanogenic populations decreased with DY addition compared to the control (*p* < 0.05). However, supplementation of DY did not affect the relative abundances of *R. albus* (*p* > 0.05), as shown in Table 5.

## 4. Discussion

### 4.1. Feed Intake and Nutrient Digestibility

Our results showed that DY supplementation consistently increased final BW, ADG, and DMI, with feed efficiency also improving compared to the control [4,32,33,34,35]. The physiological health of the animal, feed ingredients, and product type were all factors that impacted the effect of yeast supplementation on ruminant growth performance [36,37]. The addition of *S. cerevisia*e improved feed utilization efficiency [15,38], as well as OM digestion [39]. Supplementation of *S. cerevisiae* also improved rumen fiber breakdown by stimulating the growth of fibrolytic bacteria [36,40]. Dry yeast supplemented in early lactation increased DMI [39], while an increase in the pace and amount of forage NDF breakdown influenced intake and production in dairy cows. Yeast supplementation improved the abundance of fibrolytic bacteria, especially Ruminococcus and Fibrobacter, and increased fiber digestion in the rumen. These findings concurred with Uyeno et al. [41], who discovered that supplementing mid-lactation dairy cows with 10 g of ADY daily for 21 days stimulated fibrolytic bacteria in the rumen.

### 4.2. Rumen Parameters and Blood Metabolites

Low ruminal pH for extended periods of time can have a severe impact on DMI and fiber degradation, resulting in decreased productivity and considerable financial losses [36]. Pinloche et al. [6] investigated changes in ruminal bacteria in response to ADY supplementation in early lactating dairy cows. Megasphaera and Selenomonas were found to be more abundant, indicating the role of yeast in lowering lactic acid concentrations and maintaining normal ruminal pH. In this study, the ruminal pH of fattening steers was <6.0, while when adding DY, pH was maintained at >6.0. Several investigations have indicated that specific strains of ADY may be highly effective at raising and stabilizing ruminal pH [34,42].

The NH_3_-N concentration increased with DY supplementation at 10–15 g/hd/d, while the concentration of BUN was similar among treatments and ranged between 15.8 and 18.6 mg/dL in the normal range, as reported by Wanapat and Pimpa [43]. By contrast, Li et al. [5] found that the addition of yeast to the diet of dairy cows decreased BUN, while total TP was not affected, since ADY may have some potential benefit in improving nitrogen utilization. Here, DY addition had no effect on blood indicators such as blood glucose, TP, TG, and FFA. However, Geng et al. [37] noted that ADY raised serum triglyceride content in finishing bulls. Increased activity of lipolytic enzymes and greater consumption of dietary lipids by yeast preparations explain the increase in TG content [44]. The type of starch and fiber used in the diet highly affects some blood parameters [45].

### 4.3. Ruminal Volatile Fatty Acid (VFA) Profiles and Methane (CH_4_) Production

In our study, fattening steers fed DY had higher C_3_ levels, while C_2_, and the C_2_ to C_3_ ratio were lower than those in the no-supplementation group. This was attributable to increases in the lactate-utilizing bacteria *S. ruminantium* and *M. elsdenii* that convert lactate to C_3_, with yeast supplementation stimulating their growth [46]. Compared to the control, yeast supplementation increased total VFA, C_3_, and valeric acid, but decreased C_2_ and the C_2_ to C_3_ ratio. Sousa et al. [33] discovered that the molar fraction of C_3_ increased, while C_2_ was reduced in yeast supplements containing total VFA. Furthermore, the addition of yeast decreased the C_2_ to C_3_ ratio, consistent with Wang et al.’s study [47]. Yeast supplementation affected the rumen microbial community, making the environment more favorable for fiber digesting bacteria and resulting in increased amounts of individual VFA obtained in the rumen [48]. After yeast supplementation, Dias et al. [1] found a higher proportion of C_3_ in the rumen of cattle. The yeast stimulated the growth of *M. elsdenii*, which converted lactate into C_3_ and C_4_ [6,35]. However, the inclusion of yeast in the diet increased C_4_ concentration, but did not result in major changes in ruminal fermentation [49].

Propionate fermentation causes significant changes in CH_4_ in ruminants, while yeast supplementation reduces CH_4_ production. This finding agreed with those of Phesatcha et al. [7] and Wang et al. [47], who recorded that yeast supplementation reduced CH_4_ production, whereas Munoz et al. [18] found that DY supplementation had no effect on total CH_4_ emission, but tended to increase CH_4_ per unit of feed intake and CH_4_ energy output per unit of gross energy intake, while Bayat et al. [17] and Li et al. [5] discovered that yeast had no effect on CH_4_ emissions. These varied effects of yeast supplementation on CH_4_ production were attributable to the experiments’ use of different yeast strains, doses, and diets [5]. Darabighane et al. [50] showed the yeast reduced CH_4_ emissions by inducing acetogens that used more hydrogen during the C_2_ generation process. Yeast addition decreased CH_4_ emissions by increasing C_3_ production and utilizing metabolic hydrogen by acetogens to produce C_2_ [7]. In our study, the methanogenic and protozoal populations decreased with DY supplementation, with the lowest values recorded at 10–15 g/hd/d.

The microbial population was studied using real-time PCR. Results revealed that *R. flavefaciens*, *B. fibrisolvens,* and *F. succinogenes* increased, while protozoal and methanogenic populations decreased with yeast supplementation at 10–15 g/hd/d. This result concurred with findings of Sousa et al. [33], who reported that the addition of yeast significantly increased the relative population of *R. flavefaciens*. The addition of DY increased the growth of cellulolytic bacterial populations, particularly *R. flavefaciens* and *F. succinogenes*, while limiting the growth of the lactate-producing bacterium (*S. bovis*) and increasing rumen fermentation consistency [49]. Ding et al. [51] found that adding yeast enhanced the number of bacteria, fungi, protozoa, lactate-utilizing bacteria, and the rate of fiber degradation. Cellulolytic bacterial and fungal colonization in the rumen may be aided by growth factors caused by organic acids and vitamins provided by yeast. Chaucheyras-Durand et al. [36] proposed that yeast may use available oxygen on the surface of freshly ingested feeds to maintain metabolic activity, resulting in a decrease in rumen redox potential. Yeast increased microbial growth, particularly of lactic acid-utilizing bacteria, and reduced acidosis [3,33]. Han et al. [3] found yeast stabilized the ruminal pH, improved the richness of rumen microflora, and regulated rumen microflora. However, Lu et al. [52] reported that adding yeast at 6 and 12 g/d decreased CH_4_ production without affecting the number or diversity of methanogens, while Jiang et al. [46] found that the numbers of *B. fibrisolvens*, an important hemicellulolytic species, were reduced in cows supplemented with a high dose of yeast. In our study, DY supplementation lowered CH_4_ production and protozoal and methanogenic populations, with the lowest values obtained at 10–15 g/hd/d. 

## 5. Conclusions

The addition of DY at 10 g/hd/d enhanced growth performance, nutrient digestibility, rumen fermentation, and total bacterial populations while reducing protozoa and methanogenic populations and CH_4_ production in fattening steers fed with a high proportion of concentrate in the TMR diet.

## Figures and Tables

**Table 1:** Compositions of total mixed ration.

Ingredient Composition	% Dry Matter Basis
Rice straw	25.3
Corn silage	4.7
Cassava chip	26.2
Rice bran	33.4
Soybean meal	4.4
Urea	2.0
Molasses	2.0
Mineral mixture	2.0
Salt	1.0
Chemical composition	
Dry matter, %	69.8
Organic matter	91.3
Ash	9.7
Crude protein	12.8
Neutral detergent fiber	29.6
Acid detergent fiber	18.2
Total digestible nutrients (TDN) *	77.9

* Calculated value % TDN = (% digestible CP + Crude fiber (CF) + Nitrogen free extract (NFE) + (2.25 × % Digestible Ether extract (EE)).

**Table 2:** PCR primers for real-time quantitative PCR assay.

Target Species	Primer Set	Reference
Total bacteria	5′-AGCAGCCGCGGTAAT-3′ 5′-CAGGGTATCTAATCCTGTT-3′	[25]
*Fibrobacter succinogenes*	5′-GTTCGGAATTACTGGGCGTAAA-3′ 5′-CGCCTGCCCCTGAACTATC-3′	[26]
*Ruminococus flavefaciens*	5′-CGAACGGAGATAATTTGAGTTTACTTAGG-3′ 5′-CGGTCTCTGTATGTTATGAGGTATTACC-3′	[26]
*Ruminococus albus*	5′-CCCTAAAAGCAGTCTTAGTTCG-3′ 5′-CCTCCTTGCGGTTAGAACA-3′	[27]
*Butyvibrio fibrisolvens*	5′-ACCGCATAAGCGCACGGA-3′ 5′-CGGGTCCATCTTGTACCGATAAAT-3′	[28]
*Megasphera eldinii*	5′-AGATGGGGACAACAGCTGGA-3′ 5′-CGAAAGCTCCGAAGAGCCT-3′	[28]
*Methanogenic archaea*	5′-GAGGAAGGAGTGGACGACGGTA-3′ 5′-ACGGGCGGTGTGTGCAAG-3′	[29]
Protozoa	5′-GCTTTCGWTGGTAGTGTATT-3′ 5′-CTTGCCCTCYAATCGTWCT-3′	[30]

**Table 3:** Effect of yeast supplementation in high concentrate diet on average daily gain, dietary dry matter intake, feed conversion ratio, and nutrient digestibility in fattening steers.

Items	Yeast Supplementation (g/day)				SEM	Contrast	
	0	5	10	15		Linear	Quadratic
Initial body weight (BW), kg	375.5	376.1	373.8	372.6	1.21	0.14	0.43
Final BW, kg	434.4 ^a^	442.7 ^b^	462.5 ^c^	461.6 ^c^	4.85	0.04	0.52
Average daily gain, g/day	649 ^a^	744 ^b^	984 ^c^	993 ^c^	6.03	0.04	0.71
Dry matter intake, kg/day	9.2 ^b^	10.5 ^b^	11.4 ^a^	11.6 ^a^	0.13	0.04	0.29
Feed conversion (feed: gain)	14.1 ^b^	14.2 ^b^	11.6 ^a^	11.7 ^a^	0.48	0.04	0.07
Nutrient digestibility, %							
Dry matter	59.7	60.8	61.4	61.6	0.29	0.39	0.62
Organic matter	63.2 ^a^	65.6 ^b^	67.7 ^c^	67.4 ^c^	0.04	0.04	0.11
Crude protein	66.1	66.4	66.9	66.6	0.06	0.38	0.58
Neutral detergent fiber	49.8 ^a^	55.7 ^b^	57.9 ^c^	58.1 ^c^	0.04	0.04	0.04
Acid detergent fiber	45.4 ^a^	47.5 ^b^	49.9 ^c^	49.5 ^c^	0.02	0.03	0.04

^a,b,c^ means within a row with different superscripts differ significantly (*p* < 0.05); SEM = standard error of the mean.

**Table 4:** Effect of yeast supplementation in high concentrate diet on fermentation characteristics and blood metabolite in fattening steer.

Items	Yeast Supplementation (g/day)				SEM	Contrast	
	0	5	10	15		Linear	Quadratic
Ruminal pH	5.9 ^a^	6.3 ^b^	6.5 ^b^	6.5 ^b^	0.09	0.04	0.05
Temperature, °C	39.4	39.6	39.8	39.7	0.07	0.11	0.29
NH_3_-N, mg/dL	15.0 ^a^	16.9 ^b^	18.3 ^c^	18.6 ^c^	0.27	0.04	0.05
Blood metabolite, mg/dL							
Urea–Nitrogen	13.8	14.1	14.6	15.1	0.42	0.38	0.59
Glucose	66.1	67.6	66.9	67.3	1.94	0.42	0.71
Free fatty acid	0.6	0.7	0.7	0.7	0.16	0.20	0.59
Total protein	1.1	1.0	1.2	1.3	0.25	0.36	0.87
Triglyceride	0.3	0.4	0.5	0.4	0.32	0.17	0.89
Total cholesterol	4.1	4.2	4.4	4.5	0.14	0.22	0.94
Total VFAs, mmol/L	117.8	118.2	120.5	121.0	1.43	1.05	0.06
VFAs, mol/100mol							
Acetic acid (C_2_)	69.3 ^b^	68.8 ^b^	65.1 ^a^	64.6 ^a^	0.26	0.04	0.05
Propionic acid (C_3_)	22.8 ^a^	24.0 ^b^	27.2 ^c^	27.5 ^c^	0.17	0.04	0.05
Butyric acid (C_4_)	7.9	7.2	7.7	7.9	0.19	0.06	0.08
C_2_: C_3_	3.0 ^b^	2.9 ^b^	2.4 ^a^	2.3 ^a^	0.05	0.04	0.05
CH_4_ (mM/l)	28.1 ^b^	27.2 ^b^	24.9 ^a^	24.6 ^a^	0.19	0.04	0.05

^a,b,c^ means within a row with different superscripts differ significantly (*p* < 0.05); SEM = standard error of the mean; NH_3_-N = ammonia nitrogen; BUN = blood urea nitrogen; VFAs = volatile fatty acids; CH_4_ = methane production = 0.45 (C_2_) − 0.275 (C_3_) + 0.4 (C_4_) calculated according to Moss et al. [24].

**Table 5:** Effect of yeast supplementation in high concentrate diet on microbial population in fattening steers.

Items	Yeast Supplementation (g/day)				SEM	Contrast	
	0	5	10	15		Linear	Quadratic
Real-time PCR, copies/mL rumen content							
Total bacteria, ×10^9^	5.1 ^a^	6.2 ^b^	7.5 ^c^	7.9 ^c^	0.65	0.03	0.04
*F. succinogenes*, ×10^8^	2.8 ^a^	5.7 ^b^	8.2 ^c^	8.0 ^c^	0.28	0.04	0.06
*R. flavefaciens*, ×10^7^	4.8 ^a^	4.4 ^a^	6.9 ^b^	7.2 ^b^	0.44	0.04	0.07
*R. albus*, ×10^7^	3.7	3.3	3.8	3.1	0.82	0.08	0.11
*B. fibrisolvens*, ×10^6^	3.4 ^a^	5.8 ^b^	6.6 ^bc^	7.2 ^c^	0.43	0.04	0.06
*M. eldinii*, ×10^2^	1.4 ^a^	2.8 ^b^	4.1 ^c^	4.4 ^c^	0.58	0.04	0.05
Methanogens, ×10^5^	7.1 ^a^	5.4 ^b^	2.1 ^c^	2.0 ^c^	0.05	0.04	0.05
Protozoa, ×10^5^	5.6 ^a^	3.7 ^b^	1.9 ^c^	1.2 ^c^	0.18	0.03	0.04

^a,b,c^ means within a row with different superscripts differ significantly (*p* < 0.05); SEM = standard error of the mean.

## Data Availability

The data presented in this study are available on reasonable request from the corresponding author.
